# The Isolated Motor Branch of the Ulnar Nerve Injury During Open Carpal Tunnel Release

**DOI:** 10.7759/cureus.43601

**Published:** 2023-08-16

**Authors:** Wuttipong Siriwittayakorn, Warangkana Fongsri, Kraisong Watatham, Wattanai Atthakorn

**Affiliations:** 1 Department of Orthopaedics, Nakornping Hospital, Chiang Mai, THA; 2 Faculty of Medicine, Department of Orthopaedics, Prince of Songkhla University, Songkhla, THA

**Keywords:** open carpal tunnel release, carpal tunnel syndrome, ulnar nerve palsy, ulnar nerve injury, ulnar nerve, motor branch

## Abstract

A 76-year-old woman underwent open carpal tunnel release (OCTR). She had sudden sharp shooting pain in her hand, in the mid-palmar area, during the operation. She was then unable to abduct or adduct her thumb and fingers after surgery. She had no sensation impairment of the ulnar digit. The nerve conduction study confirmed a complete transection of the motor branch of the ulnar nerve (MUN). The MUN was repaired, and the patient recovered her intrinsic hand muscle function two years after the operation. The mechanism of injury, related anatomy and potentially dangerous area, clinical findings, management, and prevention are discussed.

## Introduction

Injury to the motor branch of the ulnar nerve (MUN) during open carpal tunnel release (OCTR) is extremely rare [1,2]. Such complications are considered iatrogenic injuries that should never occur during carpal tunnel surgery. A delay in definite diagnosis and treatment of MUN injury can lead to permanent loss of hand function. MUN injury during OCTR can arise from a surgeon’s inexperience, technical errors, or lack of awareness. Therefore, surgeons need to have a thorough understanding of the anatomical structures surrounding the carpal tunnel. 

## Case presentation

This study was approved by our institutional review board (reference number 037/65). A 76-year-old woman presented with carpal tunnel syndrome in her left hand. Her symptoms persisted for one year and did not improve after conservative treatments and three doses of corticosteroid injection. She underwent OCTR.

The procedure was carried out under local anesthesia with tourniquet inflation. A 1.5 cm incision was made from the distal wrist crease and continued distally along the central axis of the ring finger. The transverse carpal ligament (TCL) was cut, in the proximal to the distal direction along its ulnar-most border, just radial to the hook of the hamate. The operating surgeon tried to confirm a complete TCL transection by dissecting around the flexor tendon distal to the hook of the hamate. As he was passing the scissor under the flexor tendons, the patient felt a sharp shooting pain in the hand and yelled unpleasantly. The surgeon checked the function of her flexor tendons and the sensation of her ring and little finger; it was all intact. The procedure was then carried out till the end, and the patient was discharged.

At one month postoperative, her carpal tunnel syndrome symptoms improved. However, she presented with obvious intrinsic hand muscle atrophy. The patient complained of tolerable dull pain in the mid-palmar area. She reported a weak handgrip and loss of pinch power in her left hand at the two-week follow-up. The sensation of her ring and little finger was intact. She was concerned about her symptoms as their presence was sudden and did not improve over time.

An electrodiagnostic study was conducted to evaluate her MUN condition. There were spontaneous muscle fibrillations and positive sharp waves. The study described a sign of active denervation without reinnervation. No motor unit action potential was observed. Hence, a complete MUN transection was confirmed.

Nerve exploration was carried out about two months after the OCTR. MUN was completely divided. The injury was located in the mid-palmar space under the flexor tendons along the vertical axis of the third web space, about 1 cm distal to the hook of the hamate. We performed external neurolysis and direct nerve coaptation.

**Figure 1:**
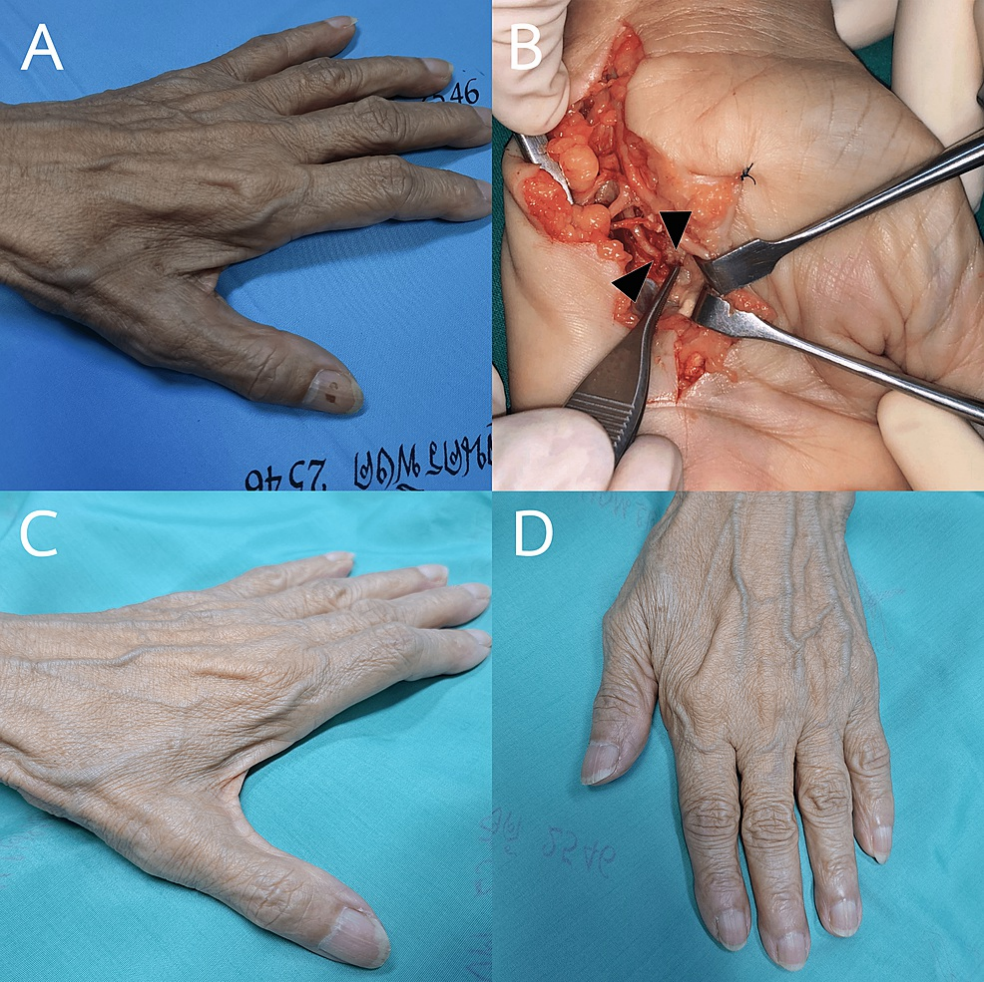
Clinical pictures of the patient. (A) The patient presented with intrinsic hand muscle atrophy one month after surgery. (B) Intraoperative findings revealed proximal and distal stumps of the nerve (indicated by black arrowhead) during microsurgical nerve repair. (C) Two years after the nerve repair, the contour of the first dorsal interosseous muscle and adductor pollicis muscle was observed. (D) The patient regained the ability to adduct her thumb and fingers again.

The patient reported an improvement in mid-palm pain one month after the nerve repair. Palpable first dorsal interosseous muscle contracture was present at eight months. She was able to abduct and adduct her thumb and fingers properly at 18 months postoperative. At 24 months after surgery, her pinch strength was 1.8 kg (right hand, 3.1 kg), handgrip strength was 7.1 kg (right hand, 15.7 kg), and the disability of arm shoulder and hand (DASH) score was 3.44. She had slight difficulties in activities that required fine finger movement, such as picking up small objects, and in activities that required a strong grip of the left hand. Otherwise, she was satisfied with the treatment result (Figure 1).

## Discussion

Ulnar nerve injury during carpal tunnel surgery is extremely rare, and an isolated MUN injury is even rarer [1,2]. Cases of isolated MUN injury during OCTR were scarcely reported. Favero and Gropper, in 1987, first described ulnar nerve main trunk injury during OCTR at the Guyon’s canal level. The carpal tunnel release incision was placed at 1 cm to the ulnar side of the center of the palm and was extended proximally over the hypothenar area [3]. The authors emphasize the importance of a well-planned skin incision and a proper understanding of the wrist anatomy before performing carpal tunnel surgery.

Terrono et al., in 1993, presented three cases of isolated MUN injury during OCTR. All injuries were not appreciated during the operation. The incision was placed either in line with the ring finger or 1 cm parallel and ulnar to the thenar crease. All injuries were identified in the mid-palmar space just distal to the hook of the hamate [4]. The author accentuated that any dissection around the hook of the hamate can propose a risk of MUN injury. The TCL should be divided in the distal to proximal direction while identifying the hook of the hamate as the ulnar distal margin.

MUN injury during OCTR was then reported again in 2008 by Yoong et al. [5]. The author placed the incision in line with the ring finger. The operation was done under local anesthesia and was complicated by the patient’s persistent bleeding. The patient reported weakness of handgrip and pinch power upon the clinical follow-up. The author found that MUN was divided at the base of the hook of the hamate. They highlighted the importance of a clear, bloodless field of carpal tunnel operation and early recognition with rapid management of the nerve injury.

**Figure 2:**
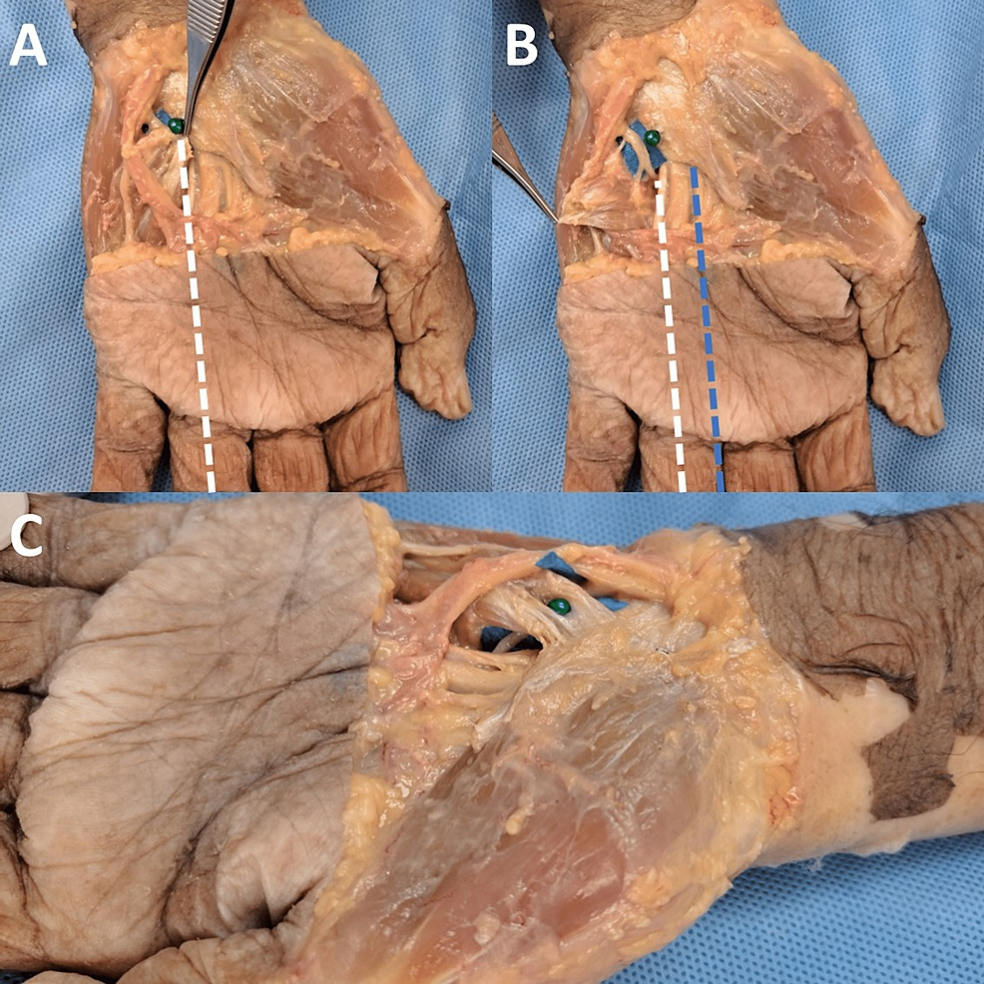
Anatomic relationship between MUN and its surrounding structures. (A) The green pin indicates the center of the palpable surface of the hook of the hamate. The central axis of the ring finger is indicated by the white dashed line. The hook of the hamate situates along the axis of the ring finger. (B) The turning point of the MUN is located distal to the hook of the hamate, aligning with the axis of the ring finger. It is safer to operate along the vertical axis of the third web space (blue dashed line) as the MUN is covered by flexor tendons in this axis. (C) The turning point of the MUN lies just below the level of the TCL before coursing under the flexor tendons. TCL, transverse carpal ligament; MUN, motor branch of the ulnar nerve

The safe zone for an incision placement in OCTR generally lies between the central axis of the ring finger and the longitudinal axis of the third web space [6,7]. However, one should note that the hook of the hamate is in line with the central axis of the ring finger [8]. As the ulnar nerve exits the Guyon's canal at the wrist, it divides into a superficial branch and a deep branch, called MUN. The deep branch courses into the muscular tunnel formed by the hypothenar muscles. MUN runs medial to the hook of the hamate and turns toward the thumb after exiting this muscular tunnel. The MUN’s turning point is about 11 mm distal to the center of the palpable surface of the hook of the hamate and situates just under the level of the TCL, along the axis of the ring finger. There could be an anatomical variation where the MUN’s turning point resides at the distal border of the hook of the hamate [9]. Every MUN injury during OCTR is associated with an incision placed on the ulnar side of the skin. Hence, placing an OCTR incision along the axis of the ring finger can propose a higher risk for MUN injury. We suggest that it is safer to perform OCTR only along the longitudinal axis of the third web space (Figure 2).

Radial to the axis of the ring finger, the MUN then turns anatomical posteriorly to the muscular floor of the palm, which is formed by the intrinsic hand muscles. It then traverses on this floor, giving branches to supply intrinsic hand muscles, and enters the adductor pollicis muscle radial to the central axis of the middle finger, under the flexor tendons [9,10]. Along the axis of the third web space, the MUN is covered by flexor tendons. The MUN is located about 35.1 and 9.2 mm from distal wrist crease and Kaplan’s cardinal line, respectively [9,11]. In our case and in all previously reported MUN injuries during OCTR, the nerve was found to be divided in the mid-palmar space. Hence, the injury could be a result of a dissection or misplacement of surgical instrument under flexor tendons distal to the TCL distal border. Thus, the area under flexor tendons should never be explored or dissected during OCTR. In addition, it would be safer if the TCL is divided from distal to proximal direction [4,12].

Injury to the MUN could be missed both during and after the OCTR [4], which could lead to severe or permanent loss of hand function when the treatment was delayed [5]. If the operation is done under regional or general anesthesia, it may be difficult to evaluate the MUN’s function during surgery. Thus, intrinsic hand muscle function should always be evaluated postoperatively after OCTR, if MUN injury was suspected intraoperatively. When surgery is done under local anesthesia, a sudden sharp shooting pain in the hand should urge the awareness of any iatrogenic injury. Likewise, intrinsic hand muscle function should be evaluated intraoperatively to identify any injury to the MUN.

When the patient presented with intrinsic hand muscle weakness and atrophy without sensory impairment of the ulnar digits, certain low ulnar nerve palsy should also be differentiated. Pure MUN compression in Guyon canal zone II can produce isolated loss of intrinsic hand muscle function. Guyon canal compression may result from number of causes, including acute or repetitive trauma, hamate hook nonunion, anomalous muscle, or space-occupying lesions [13]. However, a diagnosis of iatrogenic MUN transection is striking when the patient’s symptoms occurred acutely after the operation. Early diagnosis with electromyography and urgent microsurgical nerve repair has always been recommended in such patient to prevent the loss of function [3-5].

Clear and bloodless operation during OCTR was also highlighted to prevent MUN injury [5]. Persistent bleeding could cause frustration and complicate the operation, thus proposing a higher risk of any iatrogenic complication. Recently, postoperative MUN injury was also observed. Ashi et al. proposed that the injury could be a result of a large hematoma formation in the hand and wrist after surgery [14]. Therefore, a proper tourniquet inflation, bleeding control, and the use of local anesthetic agent with adrenaline can aid in clear and meticulous dissection [5,13]. In our practice, we always apply light wound compression to the hand and wrist with an elastic bandage after OCTR to prevent any risk of hematoma formation. The patients are advised to remove the bandage by themselves after two hours.

## Conclusions

MUN injury during OCTR is an extremely rare occurrence, but it is an iatrogenic injury that arises due to a surgeon’s inexperience, lack of awareness, or technical error. Therefore, surgeons must possess a thorough understanding of the anatomical structures surrounding the carpal tunnel before undertaking OCTR. Dissecting or exploring the area around the hook of the hamate and under the flexor tendons should be avoided. A high index of suspicion should be maintained for MUN injury when a patient presents with acute intrinsic hand muscle weakness following OCTR. In such cases, early microsurgical reconstruction is strongly recommended.
